# Screening for stress-resistance mutations in the mouse

**DOI:** 10.3389/fgene.2014.00310

**Published:** 2014-09-08

**Authors:** Wallace S. Chick, Michael Ludwig, Xiaoyun Zhao, David Kitzenberg, Kristina Williams, Thomas E. Johnson

**Affiliations:** ^1^Department of Cell and Developmental Biology, University of Colorado DenverAurora, CO, USA; ^2^Charles C. Gates Regenerative Medicine and Stem Cell Biology Program, University of Colorado DenverAurora, CO, USA; ^3^Department of Pediatrics, University of Colorado DenverAurora, CO, USA; ^4^Department of Integrative Physiology, University of Colorado BoulderBoulder, CO, USA; ^5^Institute for Behavioral Genetics, University of Colorado BoulderBoulder, CO, USA; ^6^Biofrontiers Institute, University of Colorado BoulderBoulder, CO, USA

**Keywords:** embryonic stem cells, stress resistance, mice, forward genetics, piggyBac transposon, gene-trap, paraquat

## Abstract

Longevity is correlated with stress resistance in many animal models. However, previous efforts through the boosting of the antioxidant defense system did not extend life span, suggesting that longevity related stress resistance is mediated by other uncharacterized pathways. We have developed a high-throughput platform for screening and rapid identification of novel genetic mutants in the mouse that are stress resistant. Selection for resistance to stressors occurs in mutagenized mouse embryonic stem (ES) cells, which are carefully treated so as to maintain pluripotency for mouse production. Initial characterization of these mutant ES cells revealed mutations in *Pigl, Tiam1*, and *Rffl*, among others. These genes are implicated in glycosylphosphatidylinositol biosynthesis, NADPH oxidase function, and inflammation. These mutants: (1) are resistant to two different oxidative stressors, paraquat and the omission of 2-mercaptoethanol, (2) have reduced levels of endogenous reactive oxygen species (ROS), (3) are capable of generating live mice, and (4) transmit the stress resistance phenotype to the mice. This strategy offers an efficient way to select for new mutants expressing a stress resistance phenotype, to rapidly identify the causative genes, and to develop mice for *in vivo* studies.

## Introduction

Stress resistance is a trait intimately linked to increased life span and health span in multiple species, including yeast, fruit flies, worms, and mice (Lithgow et al., 1995; Harshman and Haberer, 2000; Fabrizio et al., 2001; Johnson et al., 2002; Murakami et al., 2003). In mammals, the Ames and Snell dwarf mice are long-lived and have increased resistance toward multiple stressors, including paraquat (PQ), hydrogen peroxide, UV, and heavy metals (Salmon et al., 2005). Knocking out the growth hormone receptor (GHR) gene in mice produced a similar effect, extending the life span and concurrently exhibiting resistance to multiple forms of stress (Salmon et al., 2005). These long-lived mutants highlight the importance of the IGF-1 signaling pathway in modulating life span and in the coordinated modification of stress resistance. Interestingly, dietary restriction, a proven way to extend life span, also imparts the animal with increased resistance to multiple stresses (Masoro, 2000), possibly through a SIRT1 (Cohen et al., 2004) or mTOR (Blagosklonny, 2010) pathway. Naked mole rats, the longest-lived rodent, also appear to possess enhanced resistance to multiple stresses (Salmon et al., 2008). Although seemingly utilizing different mechanisms, these long-lived rodents converge to a common stress-resistance phenotype. These facts implicate stress resistance as a surrogate for (and perhaps as a mediator of) longevity (Perez-Campo et al., 1998; Lithgow and Walker, 2002).

Longevity genes identified in lower organisms have provided candidate orthologs for studying their impact on life span in mammals. Although this strategy yielded significant insight into the genetics of aging, it suffers from the limitation that only orthologs of mammalian genes can be identified. To overcome this bottleneck, we developed an unbiased genetic approach to identify new genes and pathways that mediate stress resistance and longevity in the mouse. Selections for increased stress resistance as a surrogate marker for extended lifespan have been performed efficiently in lower organisms (e.g., yeast, fruit flies, and nematodes) (Kennedy et al., 1995; Harshman and Haberer, 2000; De Castro et al., 2004). This approach, however, is cost-prohibitive and logistically implausible in mice. Herein, we describe our use of mouse embryonic stem (ES) cells to identify mutations that confer increased stress resistance. Novel manipulations allow these cells to become intact mice (Chick et al., 2009), which can be used for multiple tests of increased longevity and reduced rates of aging. The use of ES cells has several advantages: (1) it allows the screening of vast numbers of independent mutations for the phenotype of interest; (2) genetic tools are widely available to manipulate the genome of the cells; and (3) stress-resistant ES cells can readily be converted into living mice for whole-animal studies.

In our previous studies we demonstrated that mice derived from stress-resistant ES cells retained the stress-resistance Phenotype in tail skin fibroblast and were able to transmit increased stress resistance to their offspring (Chick et al., 2009). In these initial proof-of-principal studies, we used N-ethyl-N-nitrosourea (ENU) to generate a highly mutagenized ES cell population, which facilitated the isolation of stress-resistant mutants (Chick et al., 2009). Although the screen was successful, it suffered from two drawbacks. First, cells that had been exposed to the stressor were no longer competent to form mouse embryos; second, the ENU-induced point mutations were not capable of being mapped with sufficient resolution as to allow the mutant genes to be identified. We overcame the loss of pluripotency by developing a sib-selection method, which allowed the development of fertile adult mice (Chick et al., 2009). Herein, we have employed a transposon: *piggyBac* (*PB*) as a mutagen to facilitate gene identification. We report here the recovery of 7 *PB*-mediated, stress-resistant mutants identified via mass-selection for paraquat (PQ) resistance. These stress-resistant ES cells retained germline competence and are being converted into living mice for further in-depth investigations. The genes responsible for stress resistance are implicated in diverse functions including NADPH oxidase (Nox) activities, inflammation, and protein anchoring. These new mutants will be valuable animal models to investigate how those mutations affect stress resistance and their potential roles in extension of both life and health-span.

## Materials and methods

### ES cell culture

We generated a mouse ES cell lines with a F1 hybrid background (C57BL/6J × 129X1/SvJ), designated as *Blm^tet/tet^* C9, in which the expression of Bloom Syndrome helicase like protein (*Blm*) genes could be turned off by doxycycline (Supplemental Figure 1). The transient knock out of *Blm* will increase the rate of loss of heterozygosity, allowing for the generation of homozygous mutations in culture (Yusa et al., 2004) so that recessive mutations could be captured in our screen. C9 ES cells were cultured in ES cell medium with the composition as follow: Knock-Out DMEM (Life Technologies), 15% FBS, 1000 unit/ml of leukemia inhibitory factors (ESGRO, Millipore), 100 μM nonessential amino acids, 2 mM GlutaMAX, 55 μM 2-mercaptoethanol, and 25 unit/ml penicillin/streptomycin. With the exception for selection for stress resistance, ES cells were cultured on feeder layers of mitomycin C-inactivated primary mouse embryonic fibroblast (PMEF). We routinely passage the ES cells every other day in a ratio of 1:8–1:10.

### Cloning of *piggyBac* polyA trap vector (PB-UPA)

**Figure 1:**
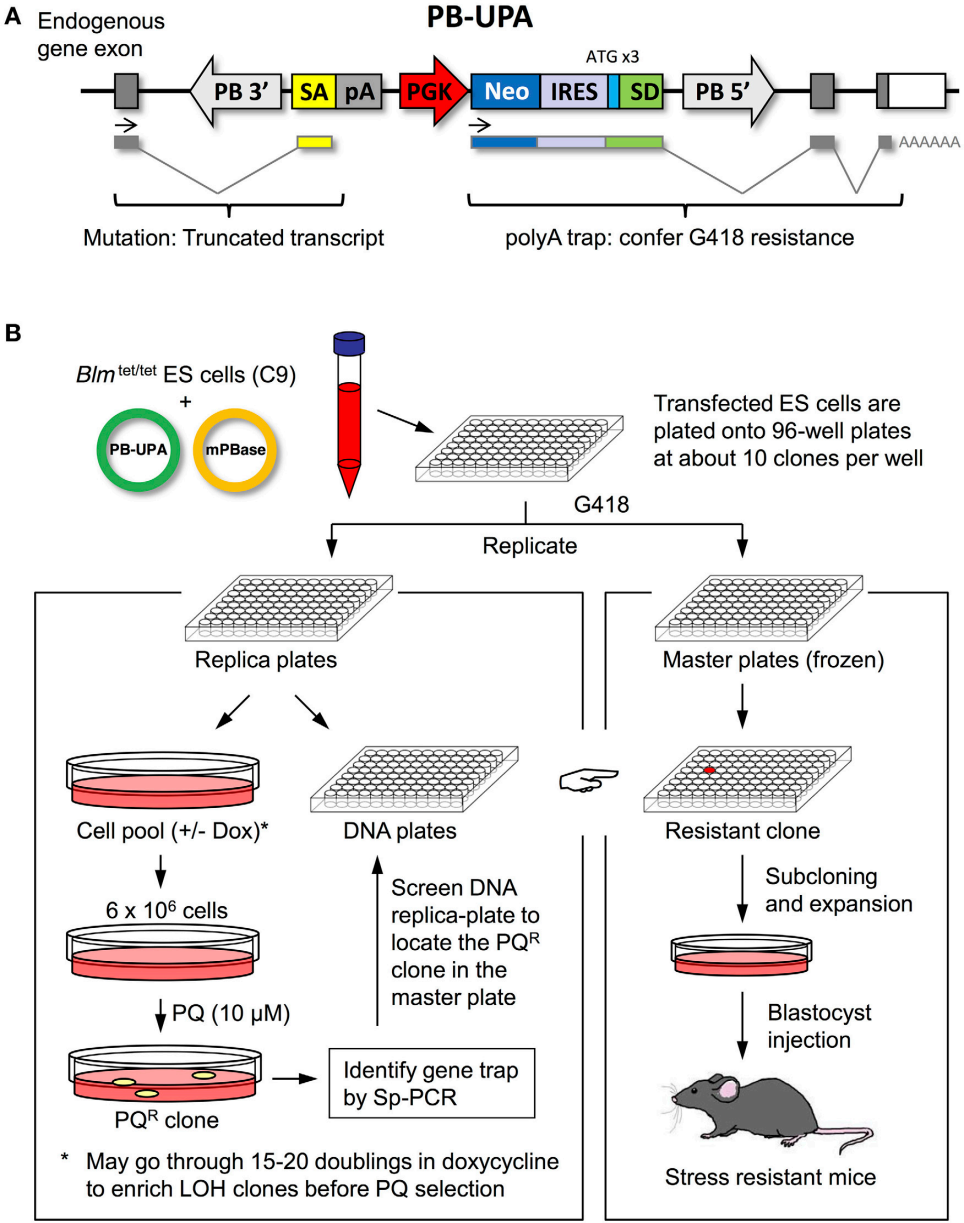
**Methods used in ES-cell mutagenesis and selection for stress resistance. (A)** Anatomy of the piggyBac (PB)-UPA vector. A UPA Trap vector containing a splice acceptor (SA), the bovine growth hormone poly-adenylate signal (pA), the phosphoglycerate kinase (PGK) promoter, the neomycin phosphotransferase gene (neo), an internal ribosomal entry site (IRES), and a splice donor (SD) with ATG (in 3 different reading frames) was cloned into the *piggyBac* transposon vector, flanked by the 3′ and 5′ long terminal repeats (PB 3′ and PB 5′) to yield PB-UPA. Gene-trapping occurs when the PB-UPA inserts into the intron of a gene. The exon upstream of the insertion site splices to the SA of PB-UPA and the transcription will be terminated by the pA. At the same time, the expression of neo, driven by PGK, will be possible when the SD splices to the downstream exons and acquires the endogenous polyA signal from the trapped gene, conferring G418 resistance to the cell and allowing selection of ES cells carrying the PB-UPA vector. **(B)** Method of mutagenesis in ES cells and selection for stress-resistant clones. ES cells were co-transfected with PB-UPA and PB transposase (mPBase) by electroporation, followed by plating into 96-well plates. Gene-trapped clones were selected by G418 resistance. Once confluent, wells were divided into a master and a replicate set. The master was frozen down and the other set was further divided into two, one for selection of resistance to paraquat (PQ) and the other for DNA isolation and gene identification. PQ-resistant cells were selected by exposure to 10 μM PQ for 7 days. Putative stress-resistant colonies were recovered and the site of PB insertion determined by Sp-PCR. Primers were then designed to screen the replica DNA library to determine the well containing the desired PQ^R^ clone, which could be subsequently recovered from the frozen master. Because each well on the master plate contains a mixture of about 9 clones, a subcloning step was performed to isolate the pure clonal line of PQ^R^ ES cells. These cells are used for various stress-resistance assays and for mouse production.

The unbiased polyA (UPA) trap vector was cloned by routine restriction digestion, or PCR, and ligation so that genetic elements were put in the order of splice-acceptor (SA) from the *Bcl2* gene, bovine growth hormone polyA signal (bGHpA), phosphoglycerate kinase (PGK) promoter, neomycin resistant cassette (neo), internal ribosomal entry site (IRES), and splice-donor (SD) from the *Hprt* gene. This UPA trap was then subcloned into pCyL50 (a gift from the Wellcome Trust Sanger Institute) at the *Xho*I site which is flanked by the 3′ and 5′ long terminal repeat (LTR) of *PB*. The resulting *piggyBac*-unbiased polyA-trap vector was designated as PB-UPA (Figure 1A).

### Mutagenesis in ES cells

C9 ES cells (5 × 10^6^) were electroporated with 1 μg of PB-UPA and 20 μg of mPB transposase (a gift from the Wellcome Trust Sanger Institute) (Cadinanos and Bradley, 2007) to generate gene-trap clones, which were selected by G418 resistance. Six electroporations were performed and pooled so as to generate approximately 9000 gene-trap clones. Transfected cells were plated evenly into ten 96-well plates which contain, on average, 9 unique gene-trap clones per well under G418 (150 μg/ml) selection. Each plate represented is a sub-library of gene-trapped clones and was handled separately. When the gene-trapped ES cells were grown to near confluency (typically after 7 days), half of the cells were frozen as the master stock. The rest were replica-plated into another 96-well plate for the isolation of DNA and selection for stress-resistant cells (Figure 1B). This replica-plating strategy allows us to preserve replica clones of ES cells that have not been through stress treatment. These protocols are crucial for maintaining pluripotency of the mutant ES cells, allowing the production of fertile mice.

### Selection for stress resistance

Prior to stress selection, gene-trapped ES cells from each sub-library were expanded on a 100-mm culture plate in the presence of doxycyline (1 μg/ml) for 12–14 days, to generate *Blm*-deficient daughter cells harboring homozygous mutations. Next, doxycycline-treated cells from each sub-library (6 × 10^6^ cells) were plated onto a 150-mm gelatinized culture dish (totally 10) and exposed to 10 μM PQ for 7 days. Colonies of surviving cells typically arose 7 days after reinstating normal culture medium; these colonies were isolated, expanded, and analyzed.

### Isolation of genomic DNA and splinkerette-PCR

Genomic DNA of stress-resistant ES cells was isolated using Qiagen's GenePure kit and splinkerette PCR was performed as described (Uren et al., 2009). PCR products, which constitute the junction fragments at the insertion site of the *PB* transposon, were cloned (TOPO-TA, Life Technologies, Carlsbad, CA) and sequenced. The DNA sequence flanking the transposon thus obtained was aligned with the mouse genome using BLAST to identify the integration site and the gene being disrupted.

### Clone isolation on the master plate

Based on the trapped gene information from the PQ-resistant ES clone, primer pairs were designed to screen genomic DNA to locate the replica ES clone on the master plate, which had not been exposed to PQ. Isolation of genomic DNA on 96-well plate was performed as described (Ramirez-Solis et al., 1992) and 2 μl of the DNA was used for PCR. A primer annealing to the upstream genomic region of the *PB* insertion site and a primer annealing to the long terminal repeat (LTR) of *PB* were employed for PCR (Supplemental Table 1A). After the well containing the replica clone of the PQ-resistant ES cells had been identified in the master plate, the entire master well of ES cells was thawed and expanded. Because each well contained a mixture of about 9 different clones, a subcloning step was performed by seeding 1000 cells onto a 100-mm culture dish followed by picking 192 individual colonies. Correct clones were then identified by PCR. These clonal lines of ES cells were used for subsequent characterization and mouse production.

### Generation of homozygous ES mutant clones

To induce homozygosity of the mutation in culture, we treated the heterozygous mutant ES cells with doxycycline (1 μg/ml) for 12–14 days, during which time the cells were passaged every 48 h. Next, 1 × 10^6^ cells were seeded onto a 100-mm culture plate containing 2 mg/ml of G418 to select homozygous clones. Surviving cells under high G418 treatment were then trypsinized and 1000 cells were plated onto another 100-mm plate. This low density of cells ensures recovery of single clones. Typically after 7 days, we hand-picked 96 individual colonies to a 96-well plate and identified homozygous clones by PCR using a 3-primer strategy (Supplemental Table 1A).

### Remobilization of *piggyBac* transposon

Inserted *PB* transposons could be remobilized by transient expression of *PB* transposase. Excision of the *PB* element causes reversion to the original wild-type DNA sequence and the associated loss of PQ^R^ would be tested. Gene-trapped ES cells (5 × 10^6^) were electroporated with 20 μg of an expression plasmid containing *PB* transposase and a puromycin-resistance cassette (mPB-Puro). Transfected ES cells were selected with puromycin (1 μg/ml) for 2 days to enrich for clones with reversion. Individual ES cell colonies were picked and analyzed for G418 sensitivity (conferred by the loss of PB-gene-trap cassette) and genotyped by PCR.

### RT-PCR

Total RNA from ES cells were isolated by Qiagen RNeasy kit. The first strand cDNA was then synthesized from the total RNA using the High Capacity cDNA Reverse Transcription kit (Applied Biosystem). Gene specific primers (Supplemental Table 1B) were then used to amplify the mutant transcript resulting from PB-UPA insertion. The PCR products were cloned and sequenced to verify the mutations in the transcripts.

### Copy number and gene expression analysis

The number of copies of *PB* inserted into PQ^R^ ES cells was assessed by qPCR using a probe that anneals to the neomycin resistant gene (neo). The expression level of the genes in these cells was assessed by reverse transcription (RT)-qPCR, using a probe set that spans two exons between which the *PB* had been inserted (Supplemental Table 2).

### Assessment of stress-resistant ES cells

ES cells (2.5 × 10^5^) were plated onto a 60-mm gelatinized culture dish with culture medium containing PQ (10 μM) or omission of 2-mercaptoethanol (a reducing agent normally required for culturing ES cells) and cultured for 48 h. After that, the total number of viable cells (those that excluded trypan blue) was counted. PQ at 10 μM kills about half of the wild-type ES cells in 48 h and is an optimal concentration for measuring stress resistance. The percentage of cells surviving stress treatment was calculated based on the total viable cells recovered divided by the total cells seeded. The parental clone C9 and a known stress-resistant clone, 4C11 (Chick et al., 2009), were included in the assay as controls.

### Reactive oxygen species (ROS) measurement

Cultured cells were trypsinized and resuspended in Hank's balanced salt solution (HBSS) containing 8 μM of 5-(and 6-)chloromethyl-2′,7′-dichlorodihydroflurorescein diacetate, acetyl ester (CM-H_2_DCFDA) (Life Technologies, Carlsbad, CA) and incubated for 15 min with shaking followed by plating onto a 96-well plate (20,000 cells/ 100 μl/well) for an additional 15 min incubation. The fluorescence signal was then measured using the well-scanning mode by a BioTek H1 fluorescence plate reader. Afterward, Hoechst 33342 dye (Life Technologies, Carlsbad, CA) was added to each well (1 μg/ml) and incubated at room temperature for 1 h to stain the total DNA, as a normalization factor for cell number. The ROS content was expressed as the ratio of relative fluorescent unit (RFU) of DCFDA to that of Hoechst.

### Generation of mice

Clones of resistant ES cells recovered from the master plates were used for embryo injection by the Transgenic and Gene Targeting core at the University of Colorado Denver. About 15–20 ES cells were injected into each individual C57BL/6 blastocyst. Fifteen blastocysts were then transferred to the uterus of a pseudopregnant female (strain ICR). Male chimera (indicated by the ES cells specific agouti coat color) recovered from the litter were mated with several young virgin C57BL/6J females to test for germ-line transmission. All animal work was approved by the IACUC.

### Fibroblast stress-resistance assay

Tail skin fibroblasts were isolated from 3-month old mice and cultured as described (Salmon et al., 2005). They were frozen at passage 3. Eight wild-type fibroblast lines and eight *Pigl*^*PB*/+^ fibroblast lines were thawed at the same time, and cultured on 60-mm plates for 24 h. After that, they were trypsinized, counted, and plated onto a 96-well plate at 15,000 cells per well (6 wells per line), and allowed to attach overnight. Cells were then exposed to 4 mM PQ for 6 h, after which normal media were reinstated for an overnight recovery. Next morning, cell viability was measured by MTT assay, with untreated cells serving as controls. Viability after PQ treatment was expressed as percentage of live cells with respect to the untreated cells.

## Results

We are interested in identifying novel mouse genes that lead to resistance to multiple stressors. When multi-stress-resistant mutant strains were selected in *C. elegans*, these strains were frequently found to slow the rate of aging and to extend life span (De Castro et al., 2004). Here we have developed a strategy for selecting similar mutations in mouse embryonic stem (ES) cells, as a means of identifying novel mouse genes that mediate both stress resistance and slowed aging. Importantly, as previously indicated, mutant ES cells are capable of developing into a whole mouse carrying the stress resistance phenotype (Chick et al., 2009), allowing for investigation of the mechanisms underlying stress resistance, as well as testing its relationship with aging and life extension.

### Mutagenesis by the *piggyBac* transposon-mediated UPA trap

In an earlier study using ENU mutagenesis to generate stress-resistant mutants, we did not attempt to identify the point mutants causing this phenotype because of the great difficulty it would entail. In this study, we employed a method by which these mutations can be rapidly identified and cloned using transposon-mediated mutagenesis. A gene-entrapment cassette was incorporated into the *PB* transposon to mutate coding genes in the mouse genome. We chose to employ a poly-A trap rather than a promoter trap because promoter trap will only trap actively transcribed genes, which is estimated to be 60% of the genome in ES cells, under normal conditions (Skarnes et al., 2004). In contrast, a poly-A trap would trap genes regardless of transcriptional activity, in theory giving us the potential to screen for mutations in all genes in the genome. A major drawback of the conventional poly-A trap is the strong bias toward trapping the last exon, appeared to be mediated by an endogenous nonsense-mediated decay (NMD) mechanism (Baker and Parker, 2004). By incorporating the unbiased poly-A trap (UPA) strategy in our PB-UPA transposon to suppresses NMD (Shigeoka et al., 2005), we were able to eliminate biased trapping.

We transfected C9 ES cells with the PB-UPA transposon (1 μg) and mPB transposase (20 μg) by electroporation. Approximately 0.03% of ES cells were stably integrated with PB-UPA, trapping a gene. Transfected ES cells were distributed evenly into ten 96-well plates and exposed to G418 to select gene-trapped clones. After 4 days of G418 selection, most of the non-trapped ES cells were killed while the surviving clones, on average about 9 clones per well, were proliferating. Each plate contained a unique set of gene-trapped clones, designated as a sub-library (C9PA-01 to C9PA10). Each was handled separately in downstream processing. The combined library contained approximately 9000 gene-trapped clones in total (Table 1). To better preserve pluripotency, this master library was frozen at early passage, soon after making a replica set. We used this replica set of cells for stress selection and mutation identification.

**Table 1:** **Generation of gene-trap library in C9 ES cells and the recovery of PQ resistant clones**.

**No. of ES cells electroporated**	**Transposon vector, PB-UPA (μg)**	**Transposase vector, mPBase (μg)**	**Replication**	**Total no. of gene-trap clones (G418^R^)**	**Total no. of PQ resistant clones**
5 × 10^6^	1	20	6	9000	7

*On average, a single transfection of 5 × 10^6^ ES cells yields 1500 gene-trap clones. We performed 6 transfections and pooled the cells to yield a total of 9000 gene-trap clones*.

### Selection for paraquat resistance

Prior to stressor treatment, replicated ES cells from each sub-library were expanded in the presence of doxycycline (dox) to generate clones of homozygous mutants. Dox-treated cells (6 × 10^6^ cells per 150-mm culture dish per sub-library in a total of 10 dishes) were cultured in the presence of PQ to isolate resistance mutants. PQ was chosen as our primary stressor because it kills cells effectively and cleanly, which facilitated the unambiguous isolation of surviving clones. Moreover, PQ was used in a similar strategy to identify longevity mutants in invertebrate species. We found that both the omission of the feeder layer and a low density of cells were crucial for the selection, otherwise non-resistant ES cells would not be killed by the stressors. After a 7-day exposure to PQ, we observed that surviving ES cell colonies, which varied in number, appeared in nine of the culture dishes. We picked at least 10 colonies from each of those dishes and a total of 96 putative stress-resistant colonies were isolated. Colonies isolated from the same dish frequently harbored the same gene-trap mutations indicating that they arose from a single clone. The observation of multiple PQ resistant (PQ^R^) sibling colonies within a given dish strongly suggests a prevalent stress-resistance phenotype associated with that clone. Since colonies isolated from the same sub-library usually contained identical mutations, we analyzed only 2 colonies from each sub-library. These PQ^R^ colonies were analyzed by Splinkerette PCR to identify the trapped gene. For two putative PQ^R^ clones isolated from two separate sub-libraries, we failed to locate the *PB* insertion site, presumably due to difficulty in PCR amplification, and they were excluded from further testing. Clones with successfully identified gene-trapped mutation were further characterized and reported here. Overall, by screening a total of 6 × 10^7^ cells (9000 unique gene trapped clones), we identified 7 mutant clones that were resistant to PQ (Table 1). Both recessive and dominant mutations could be captured at this point because daughter cells carrying homozygous mutations co-exist with the parental heterozygotes.

### Identification of the genes trapped in PQ-resistant clones

**Figure 2:**
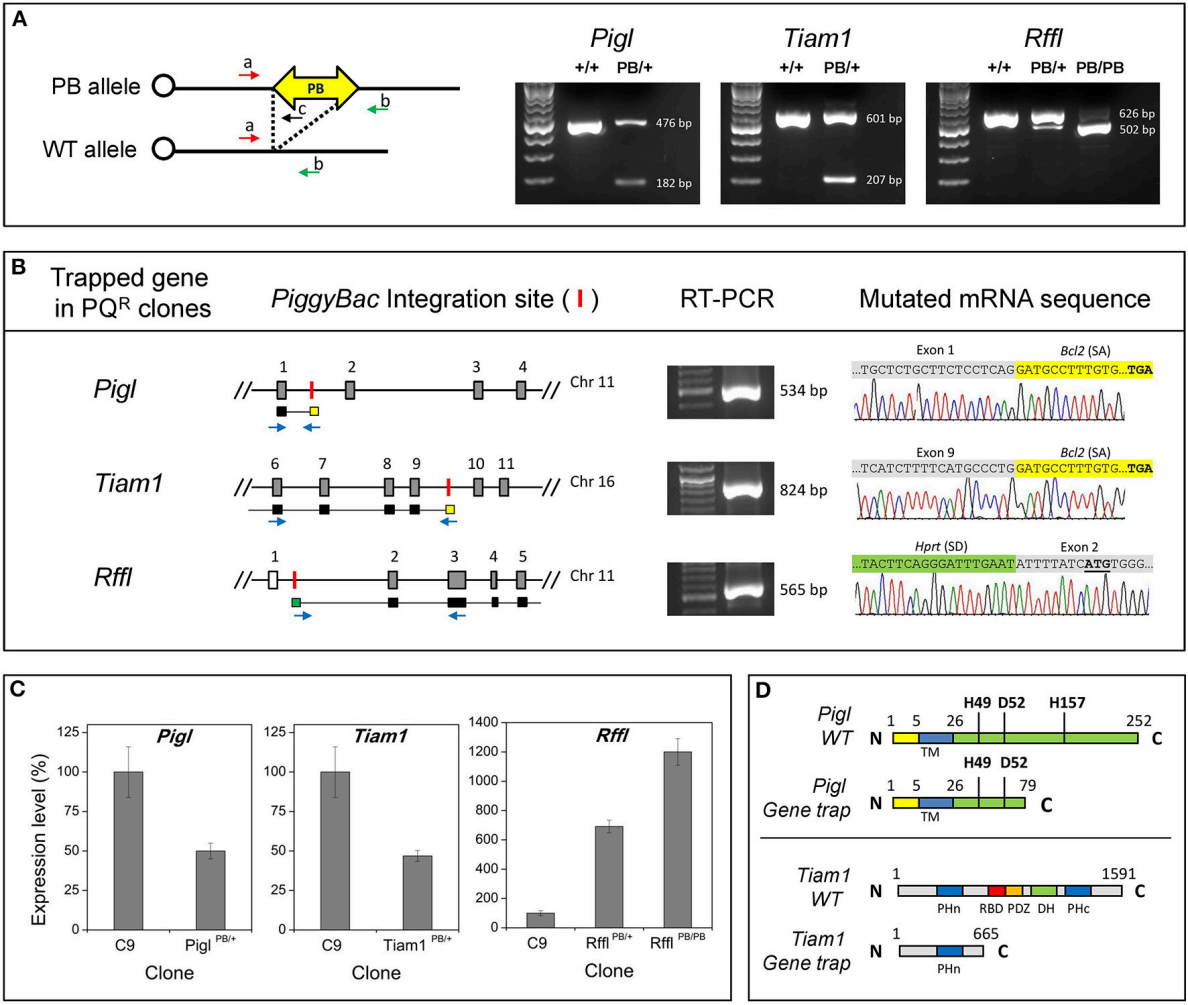
**Analysis of mutational events. (A)** Genotyping of the PB-mediated gene-trap mutants. A 3-primer methodology was used to analyze *PB* mutant clones. Flanking primers a and b will amplify an endogenous genomic fragment. However, in the PB allele, the distance between a and b is so large that no PCR product will be formed, under a short extension; instead primers a and c (annealing to the PB) will produce a product. Allele specific PCR product was visualized on an agarose gel. **(B)** Genomic location of *PB* insertion. PQ^R^ ES clones with the *PB* insertion site shown graphically (not in scale). The red vertical bar represents the site of PB insertion. Black horizontal bars under the exons represent the DNA sequence being transcribed into mRNA; the yellow box and green box represent the PB-UPA splice acceptor (SA) sequence and the splice donor (SD), respectively. The thin lines between these bars illustrate the joining of these DNA sequences. Mutant mRNA was detected by reverse transcription (RT) and PCR amplification with primer pairs as indicated by the blue arrows. These RT-PCR products were analyzed on an agarose gel showing the expected size. The DNA sequence of the RT-PCR product was determined and presented as an electropherogram showing the expected splicing events. **(C)** Gene expression level in mutant ES cells. Specific gene expression level in mutant cells was compared to the wild-type cells (C9) as measured by RT-qPCR. Error bars represent *SD* of means (*n* = 3). **(D)** Predicted protein truncation in *Pigl* and *Tiam1* mutants. Mutant Pigl protein is predicted to have the C terminal truncated, retaining 79 N-terminal amino acids. His49, Asp52, and His157 are critical residues for enzymatic activity. TM, transmembrane domain. Mutant Tiam1 protein is predicted to be a truncation retaining 665 residues at the N terminal. PH, pleckstrin homology domain; RBD, Ras-binding domain; PDZ, PSD-95 Disks large/ZO-1 homology domain; DH, Dbl-homology domain.

Trapped genes in the PQ^R^ ES cell colonies were identified by Splinkerette PCR and sequence analysis. Splinkerette PCR, although very efficient, still suffers from spurious amplification producing non-specific products. We carefully verified the putative integration site by ascertaining that the consensus TTAA site used by *PB* transposition was present in the amplified genomic DNA sequence. Junction fragments passing this criterion were used for BLAST analysis to identify the trapped genes. Seven PQ^R^ clones were identified, containing gene-trap mutations in the *Pigl, Tiam1, Rffl, Cybasc3, Ttc37* genes, as well as two uncharacterized genes, *AU019990*, and *4933439C10Rik* (Table 2). All of the *PB* integrations were found in the introns of these genes, as expected for a poly-A trap, in which the disruption of transcripts were likely caused by aberrant splicing (Figure 2B).

**Table 2:** **Genes trapped by *piggyBac* insertion in PQ resistant ES cell clones**.

**Gene-trapped**	**Chr**	**Intron**	**Gene ID**	**Nucleotide ID**	**Description**	**Function (GO)**
Pigl	11	1	ENSMUSG00000014245	NM_001039536.2	Phosphatidylinositol glycan anchor biosynthesis, class L	GPI anchor biosynthetic process
Tiam1	16	9	ENSMUSG00000002489	NM_001145886.1	T cell lymphoma invasion and metastasis 1	Regulation of Rac GTPase activity
Rffl	11	1	ENSMUSG00000020696	NM_001007465.3	Ring finger and FYVE like domain containing protein	Apoptotic process, intracellular protein transport
Cybasc3	19	3	ENSMUSG00000034445	NM_201351.1	Cytochrome b561 family, member A3	Oxidation–reduction process, lysosome
Ttc37	13	37	ENSMUSG00000033991	NM_001081352.1	Tetratricopeptide repeat domain 37	Protein binding, chaperone activity
AU019990	2	5	ENSMUSG00000074783	ENSMUST00000143964	Expressed sequence AU019990	Unknown
4933439C10Rik	11	3	ENSMUSG00000072893	ENSMUST00000132226	RIKEN cDNA 4933439C10 gene	Unknown

PQ selection and cloning of the *PB* insertion sites can be finished within a month, providing a rapid means to identify stress-resistance genes. The PQ^R^ ES cells isolated directly from stress selection, however, are not suitable for downstream characterization and mouse production. First, we are concerned that dox-induced *Blm* knock-out and exposure to PQ might have induced lesion to the cells, which would compromise phenotypic analysis. Second, exposure to stressor alone diminishes pluripotency of the ES cells resulting in the failure of mouse production (Chick et al., 2009). The current replica plating strategy addressed both of these concerns by preserving unchallenged replicate clones separately in the master plates. After the PQ^R^ clones were identified, we designed primers, specific to the trapped allele, to screen the DNA from the 96-well replica plates to locate the master wells containing the desired mutant clones. PQ^R^ gene-trapped clones thus purified were used for subsequent studies in which we focused on the *Pigl, Tiam1*, and *Rffl* mutants.

### Characterization of the *Pigl, Tiam1*, and *Rffl* gene-traps

*Pigl, Tiam1*, and *Rffl* gene-trapped ES cells were recovered from the master plate. They are morphologically indistinguishable and have a similar proliferation rate comparable to the wild-type ES cells (C9). All three clones have a single *PB* insertion, as determined by qPCR with the TaqMan probe binding to the neo, and are heterozygous with respect to the gene-trap mutations. The use of C9 ES cells containing the BLM deletion allowed us to easily generate homozygous clones in cell culture to analyze how this affects the stress-resistance phenotype. In addition, *PB* provided a molecular tag for us to easily genotype the cells by PCR using a 3-primer setup to differentiate wild-type, heterozygote, and homozygote (Figure 2A). By treating cells with doxycycline and selecting with an increased concentration of G418 (2 mg/ml), one homozygous *Rffl* clone was recovered from 96 colonies analyzed. No homozygote was isolated from the *Pigl* and *Tiam1* clones after analyzing 300 individual colonies (Supplemental Table 3). The low efficiency of LOH induction in *Pigl* and *Tiam1* may be due to a homozygous lethal phenotype. It is also possible that there is an intrinsically low level of somatic recombination in these chromosomal regions.

To confirm that the *PB* insertion causes an alteration at the mRNA level, we examined the mutant transcript by RT-PCR. In the *Pigl* and *Tiam1* mutants, as predicted, the transcripts were altered by splicing to the splice acceptor (SA) provided by PB-UPA, leading to a truncation in the encoded mRNA (Figure 2B). For the *Rffl* mutant, the PB-UPA was inserted into the intron region upstream of the initiating codon in exon 2. Sequence analysis showed that the splicing event did not alter the coding sequence of exon 2 and the downstream exons (Figure 2B); thus, the transcript is expected to be intact.

The *Pigl*^*PB*/+^ mutant showed a 50% reduction in wild-type transcript level, as determined by RT-qPCR (Figure 2C). Based on the mRNA sequence data, *PB* insertion at intron 1 of *Pigl* contributed to a truncated protein retaining 79 amino acids (out of 252) at the N-terminal (Figure 2D). The distal part of the protein containing deacetylase activity (AA44–AA167) was predicted to be lost. However, the transmembrane domain (AA5–AA26) and the cytoplasmic portion (up to AA79) that are critical for ER localization (Pottekat and Menon, 2004) were still intact. The mutant PIGL protein retained the His49 and Asp52 residues, but lost the His157 residue. These three amino acids are highly conserved and thought to be important for deacetylase activity (Urbaniak et al., 2005).

The *Tiam1*^*PB*/+^ mutant showed a 50% reduction in wild-type transcript level, determined by RT-qPCR (Figure 2C). Based on the mRNA sequence data, *Tiam1* mutants are also predicted to produce a C-terminal truncated protein. The transcription of exons upstream to the *PB* insertion produced an N-terminal portion of the protein, consisting of 665 amino acids (out of a full length of 1591 amino acid) (Figure 2D). The PHn domain, which is important for membrane location was retained but the catalytic (DH) and other domains (RBD, PDZ, PHc) (Mertens et al., 2003) were lost, suggesting the mutant *Tiam1* has likely lost all the function, as well.

When the transcript level of the *Rffl*^*PB*/+^ mutant was analyzed by RT-qPCR, a 7-fold increase of expression was observed when compared to the wild-type cells. Homozygous *Rffl^PB/PB^* mutant cells showed a 12-fold increase in expression (Figure 2C). Sequence analysis indicated that the splice donor (SD) in the PB-UPA spliced to the second exon of *Rffl*, and produced a bi-cistronic transcript driven by the PGK promoter. The mRNA sequence of *Rffl* is expected to be unchanged. The elevated expression could be explained by the stronger PGK promoter taking over the endogenous promoter to drive *Rffl* expression to a level higher than the wild-type.

### Resistance to oxidative stress

**Figure 3:**
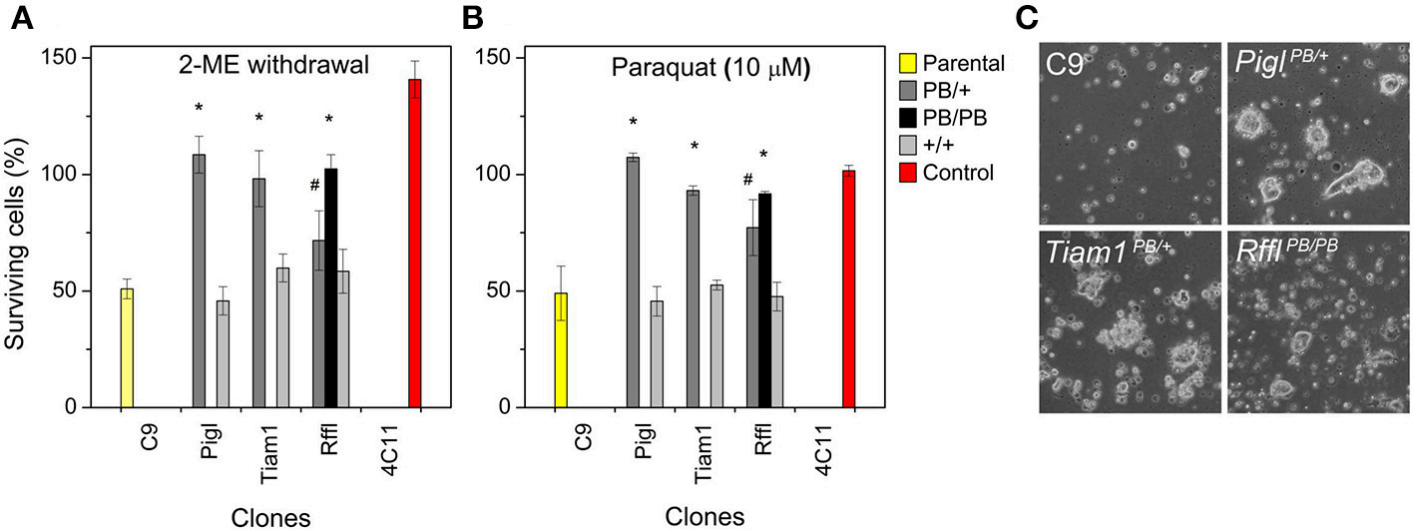
**Stress resistance in mutant ES cells. (A)** Resistance to 2-mercaptoethanol (2-ME) withdrawal. **(B)** Resistance to paraquat (PQ). Shown are the parental wild-type ES cells (C9: yellow), a stress-resistant control ES cell clone, (4C11: red), recovered from a previous study (Chick et al., 2009), and three gene-trap clones (gray). Cells were subjected to stress treatment for two days, after which the number of viable cells was counted. *Pigl, Tiam1*, and *Rffl* heterozygotes (PB/+: dark gray) exhibit resistance to both of these stressors. *Rffl* homozygotes (PB/PB: black) exhibit stronger stress resistance compared to the heterozygotes. Resistance to both stressors was lost when the PB insertions were removed (+/+: light gray). Error bars represent *SD* of mean (*n* = 4). ^*^p < 0.001, ^#^*p* = 0.01 between the gene-trap clones and the wild-type parental C9, evaluated by Student's *t*-test. **(C)** ES cell colonies formation under PQ (10 μM) treatment. Stress-resistant ES clones were able to form colonies in culture under PQ treatment while the number and size of colonies from the wild-type clone were significantly reduced.

Next, we assessed the stress resistance of the mutant ES cell clones. This test would help to confirm the resistance observed is due to the gene-trap. Given that normal ES cells are sensitive to oxidative stress, the reducing agent 2-mercaptoethanol is included in the media during routine culture. Omission of 2-mercaptoethanol kills the majority of the cells. We tested our mutant ES cells in 2 different stress conditions: (1) media without 2- mercaptoethanol, and (2) media with 10 μM PQ. Cells were cultured under stress for 2 days, after which the number of viable cells was counted. Compared to the wild-type ES cells (C9), *Pigl*^*PB*/+^, *Tiam1*^*PB*/+^, *and Rffl*^*PB*/+^ heterozygotes exhibited resistance to both of these stressors. Interestingly, *Rffl^PB/PB^* homozygotes appeared to have stronger resistance compared to its respective heterozygotes, suggesting that the level of *Rffl* expression correlates with stress resistance. When the *PB* insertions from these heterozygotes were removed, resistance to both stressors was lost (Figures 3A,B), confirming that the gene-trapping event is the causal factor for stress resistance. In culture, these resistant ES cell clones were able to form colonies under stressor treatment while non-resistant ES cells rarely did (Figure 3C). The observation of a stress-resistance phenotype in the heterozygous state suggested that all of the PQ^R^ mutations are dominant.

**Figure 4:**
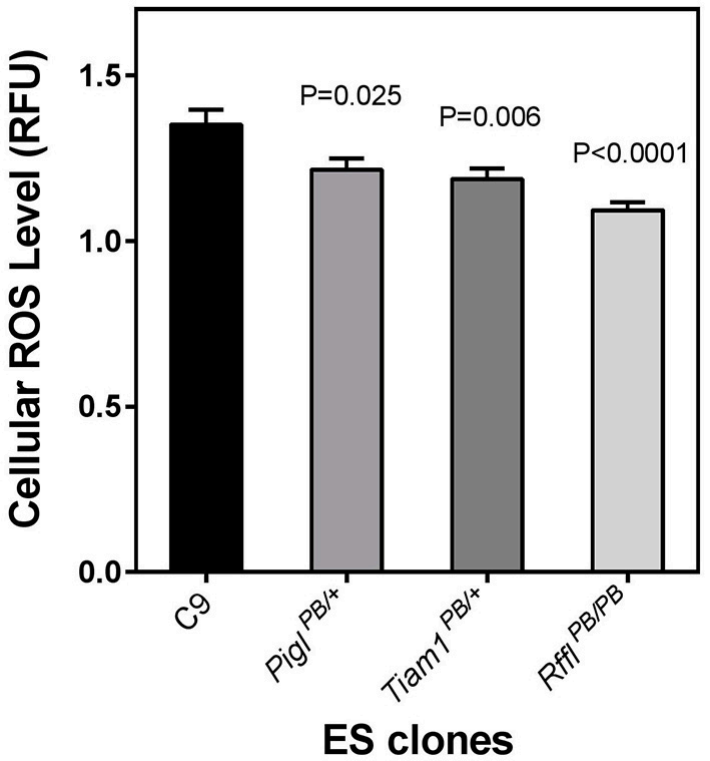
**Endogeneous reactive oxygen species (ROS) level in stress-resistant ES cells**. The ROS level of the wild-type parental C9 ES cells and three stress-resistant ES cell clones were analyzed by fluorescence spectrophotometry using the CM-H_2_DCFDA fluorescent dye normalized against Hoeshst 33342 which stained for total DNA of the cells in a given well. The fluorescence unit emitted from the CM-DCFDA was divided by the signal emitted from the DNA-bound Hoechst and expressed as a relative fluorescence unit (RFU). Error bars represent s.e.m. of means (*n* = 18). The *P* values evaluated by two tailed Student's *t*-test between the wild-type cells and each of the stress-resistant lines are indicated.

Next, we investigated whether or not the PQ^R^ phenotype may correlate with ROS level in these cells. When measuring basal cellular ROS level by CM-H_2_DCFDA, we observed all three resistant clones exhibited a lower level of ROS (Figure 4). The reduction of ROS may be a common feature of cells resistant to oxidative stresses (Brunet-Rossinni, 2004) and may also contribute to longevity (Perez-Campo et al., 1998).

### Mouse production

**Figure 5:**
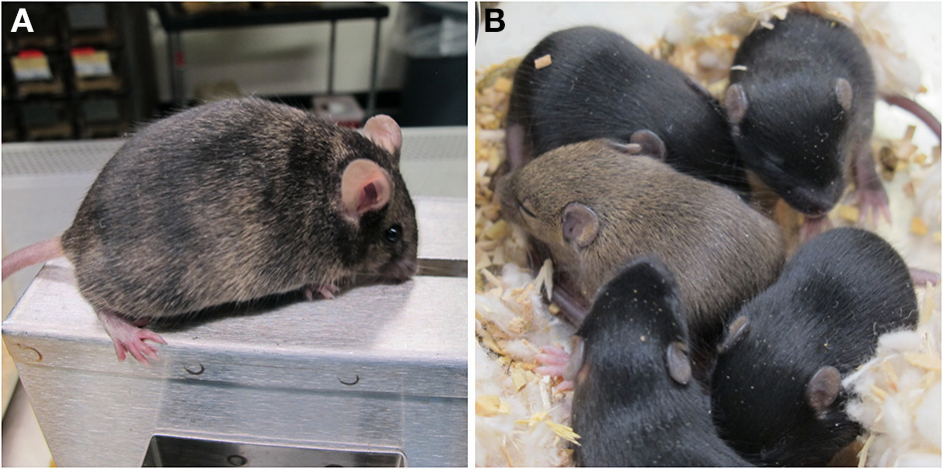
***Pigl* gene-trap mutant mice. (A)** *Pigl*^*PB*/+^ male chimera. A fertile, germline competent chimera produced by microinjection of *Pigl*^*PB*/+^ ES cells is shown. The chimerism was exhibited in the ES cell specific coat color (agouti) on a non-agouti host background (C57BL/6). **(B)** F1 pups from *Pigl*^*PB*/+^ chimera. One of the litters produced by crossing a wild-type inbred C57BL/6 female with the *Pigl*^*PB*/+^ chimera is shown. The agouti progeny indicated germline transmission. Further genotyping by PCR confirmed the inheritance of the gene-trapped *Pigl* allele.

After confirming that these gene-trapped ES cells were stress resistant, we injected the *Pigl*^*PB*/+^ and *Tiam1*^*PB*/+^ ES cells into blastocysts to generate mouse chimeras. From a single injection session, we recovered 4 chimeras of the *Pigl* mutant; two of them showed germline transmission. Two chimeras were recovered from the *Tiam1* mutant and also showed germline transmission (Supplemental Table 4). These results confirmed that PQ^R^ ES cells mutagenized by the PB-UPA mutagenesis vector retained germline competency, thus allowing the potential for production of stress-resistant mice. Both the *Pigl* and *Tiam1* chimeras and their respective F1 heterozygous pups are outwardly normal and fertile (Figure 5). Breeding is currently underway to generate a cohort of *Pigl* and *Tiam1* mice for whole animal studies.

### Recapitulation of stress-resistance phenotype in the *Pigl*^*PB*/+^ mice

**Figure 6:**
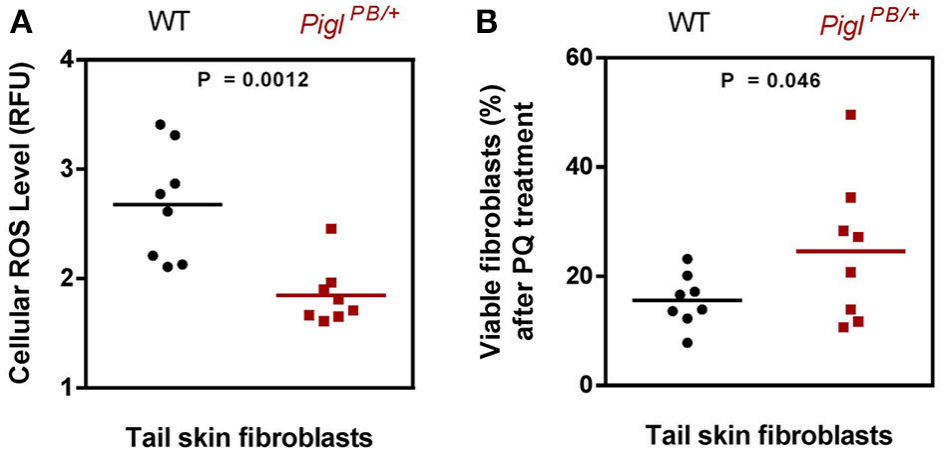
**Characterization of *Pigl*^*PB*/+^ fibroblasts. (A)** ROS level. Tail skin fibroblasts isolated from wild-type (WT) and *Pigl*^*PB*/+^ mice were stained with CM-H_2_DCFDA and the fluorescence was normalized against Hoechst. The ROS content was expressed as relative fluorescence unit (RFU). The *P* value was evaluated by two tailed Student's *t*-test (*n* = 8). **(B)** PQ resistance. Tail skin fibroblasts from wild-type (WT) and *Pigl*^*PB*/+^ mice were exposed to 4 mM PQ for 6 h, after which the viability of the cells were measured by MTT assays. The percentage of live cells after PQ treatment was calculated by the ratio of the absorbance obtained from the PQ-treated cells and that from the un-treated cells. The *P* value was evaluated by one tailed Student's *t*-test (*n* = 8). Each dot or square represent one individual mouse.

Intercross of the *Pigl*^*PB*/+^ heterozygotes consistently produced small litters (average 5 pups), from which no live homozygous *Pigl^PB/PB^* pups were identified, suggesting that homozygous *Pigl* gene-trap mutations are embryonic lethal. We have isolated tail snips from both the *Pigl*^*PB*/+^ heterozgyotes and the wild-type littermates at 3-months of age to culture fibroblasts for assessing cellular resistance to stressors. *Pigl*^*PB*/+^ fibroblasts have lower ROS content than wild-type and are more resistant to PQ-induced cell death (Figure 6). These observations indicate that the stress-resistance phenotype in ES cells was recapitulated in the mice, demonstrating the feasibility of making stress-resistant mouse by forward genetic screen in ES cells. Currently, *Pigl* mice are being investigated for several aging parameters including measurement of life span. Those studies will be presented in future communications.

## Discussion

### Significance

The use of a selectable mutant phenotype in ES cells, followed by conversion of these cells into living mice with the same phenotype, offers a novel and efficient method for performing a large-scale, high-throughput forward genetic screen to produce mouse mutants for any phenotype that can be studied in cells. A major advantage of such a screen is that it is unbiased, allowing the discovery of unexpected mutants and novel mutational events. Based on the large body of evidence that stress resistance is closely linked to life extension, both across and within species, we selected for stress-resistant ES cells as a surrogate, at the cellular level, for longevity mutants. Here we report on this novel strategy to create ES cells that are resistant to oxidative stress. Using a *piggyBac*-mediated gene-trap as the mutagen, we were able to quickly generate a total of 9000 gene-trap clones. The current collection has not yet fully captured all of the coding genes in the mouse genome (23,139 genes, based on the GRCm38 assembly on Ensembl). We envision that a full coverage can readily be achieved by accumulating mutants over repeated independent mutagenesis experiments.

The diploid nature of mammalian cells has been a limiting factor in conducting genetic studies because inactivation of one allele usually is insufficient to yield an observable phenotype due to the compensatory effect of the unaffected allele. Recessive mutations must exist in the homozygous state for them to be detected. To allow the discovery of recessive mutations causing stress resistance, we adopted the method of knocking out the Bloom syndrome helicase like protein (Blm) (Guo et al., 2004; Yusa et al., 2004; Trombly et al., 2009). Loss of Blm increases the rate of sister chromatid exchange (SCE) leading to a loss of heterozygosity (LOH) in the segment being exchanged. By culturing heterozygous mutant cells under Blm-deficient conditions, one can generate daughter cells harboring homozygous mutations. However, constitutive knock out of Blm in long-term culture leads to genome instability; thus we employed the tet-off strategy (Bond et al., 2000) to transiently turn off Blm during our genetic screen (Yusa et al., 2004).

The *piggyBac* transposon used here is superior to several other transposons (e.g., Sleeping Beauty), both for randomness and for transposition efficiency (Wu et al., 2006). It is not uncommon for a cell to receive multiple *piggyBac* insertions, which is correlated with the dose of transposon used in transfection (Wang et al., 2009). We observed that 0.03% of cells were captured by the gene-trap when transfected with 1 μg of PB-UPA. Increasing the amount of transposon DNA generally increases the number of gene-trapped clones; the fraction of cells carrying a gene trap increased to 0.18% when 5 μg of PB-UPA was used. However, the incidence of multiple transposon integrations within the same cell is also increased accordingly (Wang et al., 2009). We conclude that using 1 μg of transposon yields a reasonable number of gene-trap clones, while maintaining predominantly a single *PB* insertion per cell. To simplify genetic analysis, we have so far limited our analyses to mutants carrying a single *PB* insertion.

The use of F1 hybrid ES cells strain is another contributing factor to making this method work. Because the ultimate goal is to make stress-resistant mice from these mutant cells, the ability of the mutant ES cells to maintain pluripotency is critical. In our studies, cells need to go through multiple experimental manipulations, each of which can diminish such potential. Compared to ES cells derived from inbred strains, F1 hybrid ES cells retain exceptional developmental potential (Eggan et al., 2001), even after multiple rounds of *in vitro* manipulation such as serial gene targeting (Nyabi et al., 2009), chemical mutagenesis (Chen et al., 2000), and irradiation-induced chromosomal deletion (Chick et al., 2005). As demonstrated here, our hybrid ES cells have gone through targeted manipulations of the *Blm* gene followed by transposon-mediated gene entrapment and still maintain pluripotency, allowing mouse formation.

Generation of mutants by a polyA-trap has offered several advantages in selection for stress resistance. The polyA-trap captures both active and dormant genes in ES cells, while a promoter-trap only captures active genes. Stress-responsive genes that are not expressed in normal culture will escape from the promoter-trap and will not be identified in the screen. The inclusiveness of the polyA-trap is an important factor to consider in a screen where differential gene expression would be induced, such as the case we found here. While the promoter-trap usually induces loss-of-function, the polyA-trap could produce loss-of-function, partial loss-of-function, and gain-of-function alleles, depending on the integration site of the vector. In most cases, the poly-A trap will result in a C-terminal truncation of the protein; depending on the insertion site, the size of truncation may vary, which could produce a variety of functional outcomes. For example, a protein may retain some function even when a domain is deleted and the resulting phenotype could be different from a total loss of the same protein. To further improve the versatility of gene trapping, we eliminated the bias associated with the conventional polyA-trap, in which the last exon was preferentially trapped, by employing the UPA design. Consistent with a previous report (Shigeoka et al., 2005), we also found that our PB-UPA trap does not show such bias. In some cases, the PB-UPA trap was inserted into the first intron, resulting in a large truncation.

In this study, six out of seven PQ^R^ clones are predicted to have gene-trap mutation causing protein truncation. Quite unexpectedly, we also identified a mutation leading to an over-expression of the *Rffl* gene. This increase of expression was produced by the insertion of the PB-UPA trap in close proximity to the first coding exon, thereby replacing the endogenous promoter with the strong PGK promoter provided by the gene-trap cassette. This example illustrates that the PB-UPA trap is quite versatile in inducing multiple forms of mutations, making it the vector of choice for mutagenesis and multi-allelic analysis.

### Mutant gene ontology

The seven clones of stress-resistant ES cells recovered from the screen have mutations in genes of diverse functions. Two of them are novel genes without information on function in the literature. We focused on three mutants with mutations in known genes.

The T cell lymphoma invasion and metastasis inducing protein 1 (*Tiam1*) knock out was first shown to reduce skin tumorigenesis in mice (Malliri et al., 2002). Subsequent studies showed that the *Tiam1* knock out reduces the level of intracellular reactive oxygen species (ROS) (Rygiel et al., 2008). *Tiam1* activates RAC1, a subunit of the NADPH oxidase complex (Nox1, Nox2, and Nox3 isoforms), and in turn mediates ROS generation. The Nox family of enzymes is one of the major sources of ROS in a variety of cell types, and has recently been shown to have strong implications in inducing oxidative stress and damage. Many studies suggested that Nox activities could contribute to oxidative stress in various tissues, including the microglia (Qin et al., 2013), the vasculature (Lassegue and Griendling, 2010), liver (Kono et al., 2000), and the lung (Sato et al., 2002). Furthermore, Nox-dependent superoxide production can contribute to atherosclerosis (Judkins et al., 2010) and Hungtinton's disease (Valencia et al., 2013). In contrast, inhibiting Nox activity appears to provide protection against oxidative-stress-induced damage in neurons during cerebral ischemia (Kleinschnitz et al., 2010), in cardiac myocytes during pressure overload (Kuroda et al., 2010), and in mouse proximal tubule (MPT) cells under high glucose challenge (Sedeek et al., 2010). These studies highlight the importance of Nox in modulating oxidative stress in a broad range of tissues and the onset and progression of age-related disease. Consistent with data previously reported (Rygiel et al., 2008), we observed reduced ROS level in the *Tiam1*^*PB*/+^ ES cells, presumably through the reduction of Nox activities. The capture of the *Tiam1* mutant, a regulator of Nox, illustrates that our screen is successful in identifying a relevant genes that modulate the oxidative state of the cells.

Another hit was *Pigl* (phosphatidylinositol glycan anchor biosynthesis, class L). This gene encodes for an enzyme catalyzing the second step of the biosynthesis of the glycosylphosphatidylinositol (GPI) anchor. PIGL catalyzes the deacetylation of N-acetylglucosaminyl-phosphatidylinositol at the cytosolic side of endoplasmic reticulum (ER). The deacetylated glucosaminyl-phosphatidylinositol is flipped into the lumen of ER for further maturation to a full GPI anchor and transferred to proteins that have a GPI attachment signal at the C terminus by GPI transamidase. GPI anchored proteins are then transported to plasma membranes and membranes of cellular organelles. *PIGL* mutations, causing GPI deficiency, lead to the CHIME syndrome (Ng et al., 2012), a collective systemic disorders characterized by colobomas, congenital heart defects, early onset migratory ichthyosiform dermatosis, mental retardation, and ear anomlies. Patients with CHIME syndrome (a recessive trait) commonly have a homozygous Leu167Pro mutation, in exon 5 of the PIGL gene; heterozygotes have no disease phenotype. The mouse *Pigl*^*PB*/+^ mutant ES cells we recovered have a predicted early termination after exon 1, producing a putative truncated PIGL protein. However, the N-terminal portion of the mutant protein is still localized to the ER. In the heterozygous state, we did not observe global GPI deficiency in these cells as measured by fluorescent aerolysin (FLARE) staining using flow cytometry (data not shown). The stress-resistance phenotype in *Pigl*^*PB*/+^ ES cells may be mediated by a modification of the composition of GPI-anchored proteins on the plasma membrane or the secretion of a subset of proteins without proper attachment of the GPI anchor (Murakami et al., 2012). The possibility of protein secretion from these *Pigl*^*PB*/+^ ES cells is of particular interest because we observed that occasionally these *Pigl*^*PB*/+^ ES cells can rescue surrounding non-resistant cells from PQ treatment (unpublished observation). We also observed ROS reduction in *Pigl*^*PB*/+^ ES cells but how the *Pigl* mutation mediate such an effect is currently unknown.

Rififylin (*Rffl*) (also known as *Carp-2*) encodes a RING domain-containing ubiquitin protein ligase (E3), a potential negative regulator of TNF-induced NF-kB activation. *Rffl* targets receptor interacting protein (RIP) for ubiquitination and degradation resulting in a downregulation of NF-kB transcriptional activity so as to prevent sustained inflammatory reaction (Liao et al., 2008). The *Rffl^PB/PB^* ES cells have a 12-fold increase of *Rffl* expression; we speculate that overexpression of Rffl might decrease NF-kB activity which was suggested by Liao et al., and as such, might attenuate TNFα-dependent inflammatory response and apoptosis. However, while increasing *Rffl* expression appeared to reduce NF-kB activity, knocking out of *Rffl* did not alter the induction of NF-kB by TNFα (Ahmed et al., 2009). The precise role of *Rffl* in modulating NF-kB dependent inflammatory response remains to be clarified.

In summary, we illustrate the generation and isolation of mutant ES cells resistant to oxidative stresses via a novel forward-genetic approach. This phenotype-driven screen in ES cells has been successful in isolating stress-resistant mutants. Initial interrogation of the mutated genes suggests some of them have known physiological roles in modulating stress responses (e.g., ROS level and inflammatory state). These newly identified genes offers considerable promise as novel longevity genes in the mouse. Although we have only demonstrated the use of PQ for selection, we believe this strategy is useful in identifying novel genetic factors that confer resistance to virtually any stressor such as ER stress, heat shock, as well as cytotoxic stress such as heavy-metal exposure. Thus, this forward genetic screen has broad implication in a many field of study. Current efforts in our lab focus on generating additional PB-UPA trap libraries covering the whole genome and to exhaustively screen for additional stress-resistant mutants. We are also generating mice from these stress-resistant ES cells for whole-animal studies.

## Author contributions

Wallace S. Chick, Michael Ludwig, David Kitzenberg, and Thomas E. Johnson conceived of the experiments. Wallace S. Chick, Michael Ludwig, David Kitzenbery, Xiaoyun Zhao, and Kristina Williams performed the experiments. Wallace S. Chick and Thomas E. Johnson wrote the manuscript with input from Michael Ludwig, David Kitzenberg, and Kristina Williams.

### Conflict of interest statement

The authors declare that the research was conducted in the absence of any commercial or financial relationships that could be construed as a potential conflict of interest.

## References

[B1] Ahmed A. U. Moulin M. Coumailleau F. Wong W. W. Miasari M. Carter H. (2009). CARP2 deficiency does not alter induction of NF-kappaB by TNFalpha. Curr. Biol. 19, R15–R17; author reply R17–R19. 10.1016/j.cub.2008.11.040 19138581

[B2] Baker K. E. Parker R. (2004). Nonsense-mediated mRNA decay: terminating erroneous gene expression. Curr. Opin. Cell Biol. 16, 293–299 10.1016/j.ceb.2004.03.003 15145354

[B3] Blagosklonny M. V. (2010). Calorie restriction: decelerating mTOR-driven aging from cells to organisms (including humans). Cell Cycle 9, 683–688 10.4161/cc.9.4.10766 20139716

[B4] Bond C. T. Sprengel R. Bissonnette J. M. Kaufmann W. A. Pribnow D. Neelands T. (2000). Respiration and parturition affected by conditional overexpression of the Ca2+-activated K+ channel subunit, SK3. Science 289, 1942–1946 10.1126/science.289.5486.1942 10988076

[B5] Brunet-Rossinni A. K. (2004). Reduced free-radical production and extreme longevity in the little brown bat (Myotis lucifugus) versus two non-flying mammals. Mech. Ageing Dev. 125, 11–20 10.1016/j.mad.2003.09.003 14706233

[B6] Cadinanos J. Bradley A. (2007). Generation of an inducible and optimized piggyBac transposon system. Nucleic Acids Res. 35:e87 10.1093/nar/gkm446 17576687 PMC1919496

[B7] Chen Y. Yee D. Dains K. Chatterjee A. Cavalcoli J. Schneider E. (2000). Genotype-based screen for ENU-induced mutations in mouse embryonic stem cells. Nat. Genet. 24, 314–317 10.1038/73557 10700191

[B8] Chick W. S. Drechsel D. A. Hammond W. Patel M. Johnson T. E. (2009). Transmission of mutant phenotypes from ES cells to adult mice. Mamm. Genome 20, 734–740 10.1007/s00335-009-9228-z 19795169 PMC2809776

[B9] Chick W. S. Mentzer S. E. Carpenter D. A. Rinchik E. M. Johnson D. You Y. (2005). X-ray-induced deletion complexes in embryonic stem cells on mouse chromosome 15. Mamm. Genome 16, 661–671 10.1007/s00335-005-0011-5 16245023

[B10] Cohen H. Y. Miller C. Bitterman K. J. Wall N. R. Hekking B. Kessler B. (2004). Calorie restriction promotes mammalian cell survival by inducing the SIRT1 deacetylase. Science 305, 390–392 10.1126/science.1099196 15205477

[B11] De Castro E. Hegi De Castro S. Johnson T. E. (2004). Isolation of long-lived mutants in *Caenorhabditis elegans* using selection for resistance to juglone. Free Radic. Biol. Med. 37, 139–145 10.1016/j.freeradbiomed.2004.04.021 15203185

[B12] Eggan K. Akutsu H. Loring J. Jackson-Grusby L. Klemm M. Rideout W. M. 3rd. (2001). Hybrid vigor, fetal overgrowth, and viability of mice derived by nuclear cloning and tetraploid embryo complementation. Proc. Natl. Acad. Sci. U.S.A. 98, 6209–6214 10.1073/pnas.101118898 11331774 PMC33447

[B13] Fabrizio P. Pozza F. Pletcher S. D. Gendron C. M. Longo V. D. (2001). Regulation of longevity and stress resistance by Sch9 in yeast. Science 292, 288–290 10.1126/science.1059497 11292860

[B14] Guo G. Wang W. Bradley A. (2004). Mismatch repair genes identified using genetic screens in Blm-deficient embryonic stem cells. Nature 429, 891–895 10.1038/nature02653 15215866

[B15] Harshman L. G. Haberer B. A. (2000). Oxidative stress resistance: a robust correlated response to selection in extended longevity lines of Drosophila melanogaster? J. Gerontol. A Biol. Sci. Med. Sci. 55, B415–B417 10.1093/gerona/55.9.B415 10995037

[B16] Johnson T. E. Henderson S. Murakami S. De Castro E. De Castro S. H. Cypser J. (2002). Longevity genes in the nematode *Caenorhabditis elegans* also mediate increased resistance to stress and prevent disease. J. Inherit. Metab. Dis. 25, 197–206 10.1023/A:1015677828407 12137228

[B17] Judkins C. P. Diep H. Broughton B. R. Mast A. E. Hooker E. U. Miller A. A. (2010). Direct evidence of a role for Nox2 in superoxide production, reduced nitric oxide bioavailability, and early atherosclerotic plaque formation in ApoE-/- mice. Am. J. Physiol. Heart Circ. Physiol. 298, H24–H32 10.1152/ajpheart.00799.2009 19837950

[B18] Kennedy B. K. Austriaco N. R. Jr. Zhang J. Guarente L. (1995). Mutation in the silencing gene SIR4 can delay aging in *S. cerevisiae*. Cell 80, 485–496 10.1016/0092-8674(95)90499-9 7859289

[B19] Kleinschnitz C. Grund H. Wingler K. Armitage M. E. Jones E. Mittal M. (2010). Post-stroke inhibition of induced NADPH oxidase type 4 prevents oxidative stress and neurodegeneration. PLoS Biol. 8:e1000479 10.1371/journal.pbio.1000479 20877715 PMC2943442

[B20] Kono H. Rusyn I. Yin M. Gabele E. Yamashina S. Dikalova A. (2000). NADPH oxidase-derived free radicals are key oxidants in alcohol-induced liver disease. J. Clin. Invest. 106, 867–872 10.1172/JCI9020 11018074 PMC517812

[B21] Kuroda J. Ago T. Matsushima S. Zhai P. Schneider M. D. Sadoshima J. (2010). NADPH oxidase 4 (Nox4) is a major source of oxidative stress in the failing heart. Proc. Natl. Acad. Sci. U.S.A. 107, 15565–15570 10.1073/pnas.1002178107 20713697 PMC2932625

[B22] Lassegue B. Griendling K. K. (2010). NADPH oxidases: functions and pathologies in the vasculature. Arterioscler. Thromb. Vasc. Biol. 30, 653–661 10.1161/ATVBAHA.108.181610 19910640 PMC2841695

[B23] Liao W. Xiao Q. Tchikov V. Fujita K. Yang W. Wincovitch S. (2008). CARP-2 is an endosome-associated ubiquitin ligase for RIP and regulates TNF-induced NF-kappaB activation. Curr. Biol. 18, 641–649 10.1016/j.cub.2008.04.017 18450452 PMC2587165

[B24] Lithgow G. J. Walker G. A. (2002). Stress resistance as a determinate of C. elegans lifespan. Mech. Ageing Dev. 123, 765–771 10.1016/S0047-6374(01)00422-5 11869734

[B25] Lithgow G. J. White T. M. Melov S. Johnson T. E. (1995). Thermotolerance and extended life-span conferred by single-gene mutations and induced by thermal stress. Proc. Natl. Acad. Sci. U.S.A. 92, 7540–7544 10.1073/pnas.92.16.7540 7638227 PMC41375

[B26] Malliri A. Van Der Kammen R. A. Clark K. Van Der Valk M. Michiels F. Collard J. G. (2002). Mice deficient in the Rac activator Tiam1 are resistant to Ras-induced skin tumours. Nature 417, 867–871 10.1038/nature00848 12075356

[B27] Masoro E. J. (2000). Caloric restriction and aging: an update. Exp. Gerontol. 35, 299–305 10.1016/S0531-5565(00)00084-X 10832051

[B28] Mertens A. E. Roovers R. C. Collard J. G. (2003). Regulation of Tiam1-Rac signalling. FEBS Lett. 546, 11–16 10.1016/S0014-5793(03)00435-6 12829230

[B29] Murakami S. Salmon A. Miller R. A. (2003). Multiplex stress resistance in cells from long-lived dwarf mice. FASEB J. 17, 1565–1566 10.1096/fj.02-1092fje 12824282

[B30] Murakami Y. Kanzawa N. Saito K. Krawitz P. M. Mundlos S. Robinson P. N. (2012). Mechanism for release of alkaline phosphatase caused by glycosylphosphatidylinositol deficiency in patients with hyperphosphatasia mental retardation syndrome. J. Biol. Chem. 287, 6318–6325 10.1074/jbc.M111.331090 22228761 PMC3307314

[B31] Ng B. G. Hackmann K. Jones M. A. Eroshkin A. M. He P. Wiliams R. (2012). Mutations in the glycosylphosphatidylinositol gene PIGL cause CHIME syndrome. Am. J. Hum. Genet. 90, 685–688 10.1016/j.ajhg.2012.02.010 22444671 PMC3322218

[B32] Nyabi O. Naessens M. Haigh K. Gembarska A. Goossens S. Maetens M. (2009). Efficient mouse transgenesis using Gateway-compatible ROSA26 locus targeting vectors and F1 hybrid ES cells. Nucleic Acids Res. 37:e55 10.1093/nar/gkp112 19279185 PMC2673446

[B33] Perez-Campo R. Lopez-Torres M. Cadenas S. Rojas C. Barja G. (1998). The rate of free radical production as a determinant of the rate of aging: evidence from the comparative approach. J. Comp. Physiol. B 168, 149–158 10.1007/s003600050131 9591361

[B34] Pottekat A. Menon A. K. (2004). Subcellular localization and targeting of N-acetylglucosaminyl phosphatidylinositol de-N-acetylase, the second enzyme in the glycosylphosphatidylinositol biosynthetic pathway. J. Biol. Chem. 279, 15743–15751 10.1074/jbc.M313537200 14742432

[B35] Qin L. Liu Y. Hong J. S. Crews F. T. (2013). NADPH oxidase and aging drive microglial activation, oxidative stress, and dopaminergic neurodegeneration following systemic LPS administration. Glia 61, 855–868 10.1002/glia.22479 23536230 PMC3631289

[B36] Ramirez-Solis R. Rivera-Perez J. Wallace J. D. Wims M. Zheng H. Bradley A. (1992). Genomic DNA microextraction: a method to screen numerous samples. Anal. Biochem. 201, 331–335 10.1016/0003-2697(92)90347-A 1632522

[B37] Rygiel T. P. Mertens A. E. Strumane K. Van Der Kammen R. Collard J. G. (2008). The Rac activator Tiam1 prevents keratinocyte apoptosis by controlling ROS-mediated ERK phosphorylation. J. Cell Sci. 121, 1183–1192 10.1242/jcs.017194 18349077

[B38] Salmon A. B. Murakami S. Bartke A. Kopchick J. Yasumura K. Miller R. A. (2005). Fibroblast cell lines from young adult mice of long-lived mutant strains are resistant to multiple forms of stress. Am. J. Physiol. Endocrinol. Metab. 289, E23–E29 10.1152/ajpendo.00575.2004 15701676

[B39] Salmon A. B. Sadighi Akha A. A. Buffenstein R. Miller R. A. (2008). Fibroblasts from naked mole-rats are resistant to multiple forms of cell injury, but sensitive to peroxide, ultraviolet light, and endoplasmic reticulum stress. J. Gerontol. A Biol. Sci. Med. Sci. 63, 232–241 10.1093/gerona/63.3.232 18375872 PMC2710579

[B40] Sato K. Kadiiska M. B. Ghio A. J. Corbett J. Fann Y. C. Holland S. M. (2002). *In vivo* lipid-derived free radical formation by NADPH oxidase in acute lung injury induced by lipopolysaccharide: a model for ARDS. FASEB J. 16, 1713–1720 10.1096/fj.02-0331com 12409313

[B41] Sedeek M. Callera G. Montezano A. Gutsol A. Heitz F. Szyndralewiez C. (2010). Critical role of Nox4-based NADPH oxidase in glucose-induced oxidative stress in the kidney: implications in type 2 diabetic nephropathy. Am. J. Physiol. Renal Physiol. 299, F1348–F1358 10.1152/ajprenal.00028.2010 20630933

[B42] Shigeoka T. Kawaichi M. Ishida Y. (2005). Suppression of nonsense-mediated mRNA decay permits unbiased gene trapping in mouse embryonic stem cells. Nucleic Acids Res. 33:e20 10.1093/nar/gni022 15687378 PMC548380

[B43] Skarnes W. C. Von Melchner H. Wurst W. Hicks G. Nord A. S. Cox T. (2004). A public gene trap resource for mouse functional genomics. Nat. Genet. 36, 543–544 10.1038/ng0604-543 15167922 PMC2716026

[B44] Trombly M. I. Su H. Wang X. (2009). A genetic screen for components of the mammalian RNA interference pathway in Bloom-deficient mouse embryonic stem cells. Nucleic Acids Res. 37:e34 10.1093/nar/gkp019 19223321 PMC2651804

[B45] Urbaniak M. D. Crossman A. Chang T. Smith T. K. Van Aalten D. M. Ferguson M. A. (2005). The N-acetyl-D-glucosaminylphosphatidylinositol De-N-acetylase of glycosylphosphatidylinositol biosynthesis is a zinc metalloenzyme. J. Biol. Chem. 280, 22831–22838 10.1074/jbc.M502402200 15817455

[B46] Uren A. G. Mikkers H. Kool J. Van Der Weyden L. Lund A. H. Wilson C. H. (2009). A high-throughput splinkerette-PCR method for the isolation and sequencing of retroviral insertion sites. Nat. Protoc. 4, 789–798 10.1038/nprot.2009.64 19528954 PMC3627465

[B47] Valencia A. Sapp E. Kimm J. S. McClory H. Reeves P. B. Alexander J. (2013). Elevated NADPH oxidase activity contributes to oxidative stress and cell death in Huntington's disease. Hum. Mol. Genet. 22, 1112–1131 10.1093/hmg/dds516 23223017 PMC3578411

[B48] Wang W. Bradley A. Huang Y. (2009). A piggyBac transposon-based genome-wide library of insertionally mutated Blm-deficient murine ES cells. Genome Res. 19, 667–673 10.1101/gr.085621.108 19233961 PMC2665785

[B49] Wu S. C. Meir Y. J. Coates C. J. Handler A. M. Pelczar P. Moisyadi S. (2006). piggyBac is a flexible and highly active transposon as compared to sleeping beauty, Tol2, and Mos1 in mammalian cells. Proc. Natl. Acad. Sci. U.S.A. 103, 15008–15013 10.1073/pnas.0606979103 17005721 PMC1622771

[B50] Yusa K. Horie K. Kondoh G. Kouno M. Maeda Y. Kinoshita T. (2004). Genome-wide phenotype analysis in ES cells by regulated disruption of Bloom's syndrome gene. Nature 429, 896–899 10.1038/nature02646 15215867

