# Supplementary material for: Screening for stress-resistance mutations in the mouse
Source: Front Genet. 2014 Sep 8;5:310. doi: 10.3389/fgene.2014.00310 (PMC4157564; doi:10.3389/fgene.2014.00310)
Supplement: Supplementary file 1 [file DataSheet1.DOCX]

**Supplemental Table 1A.** PCR Primers used for the identification of *PB* insertion site in stress-resistant ES cell clones.

| **Gene-trapped clone** | **Forward primer** | **Reverse primer** | **Amplicon size (bp) and origin*** |
| --- | --- | --- | --- |
| *Pigl* | Pigl-I1-F2  5’-acccacaccccagtcctttagt-3’ | PB3’-seq  5’-ttttacgcatgattatctttaacgtacgtc-3’ | 182 [PB] |
|  |  | Pigl-I1-R2  5’-tgcatagcagcacacacaaatg-3’ | 476 [WT] |
| *Tiam1* | Tiam1-I9-F1  5’-tgcttttcttccctcctgagac-3’ | PB3’-seq  5’-ttttacgcatgattatctttaacgtacgtc-3’ | 207 [PB] |
|  |  | Tiam1-I9-R1  5’-cccatatctgtggattcaagca-3’ | 601 [WT] |
| *Rffl* | Rffl-I1-F1  5’-gtcctggcctgttctgctatgt-3’ | PB3’-seq  5’-ttttacgcatgattatctttaacgtacgtc-3’ | 502 [PB] |
|  |  | Rffl-I1-R1  5’-cactccccaccacacctcttac-3’ | 626 [WT] |
| *Cybasc3* | Cybasc3-I3-F1  5’-cttcacacatcattccctctgg-3’ | PB3’-2  5’-atatacagaccgataaaacacatgcgtcaa-3’ | 414 [PB] |
| *Ttc37* | Ttc37-I31-F1  5’-aatcttgtccctgcagatgtga-3’ | PB3’-2  5’-atatacagaccgataaaacacatgcgtcaa-3’ | 260 [PB] |
| *AU019990* | AU019990-I5-F1  5’-tcagcatcaaaaggatgaatgg-3’ | PB3’-2  5’-atatacagaccgataaaacacatgcgtcaa-3’ | 346 [PB] |
| *4933439C10Rik* | C10Rik-I3-F1  5’-tttagatccttttggccagctc-3’ | PB3’-2  5’-atatacagaccgataaaacacatgcgtcaa-3’ | 241 [PB] |

* The PCR products amplified from the wild-type and *piggyBac* tagged allele are denoted as [WT] and [PB], respectively.

**Supplemental Table 1B.** PCR Primers used for the amplification of the mutant transcript after first strand cDNA synthesis.

| Gene-trapped clone | Forward primer | Reverse primer | Amplicon size (bp) |
| --- | --- | --- | --- |
| *Pigl* | Pigl-E1-F1  5’-attggtgggtttcttgtgtgtg-3’ | HA-R1  5’-gtagtctggcacgtcgtaaggata-3’ | 534 |
| *Tiam1* | Tiam1-E6-F1  5’-gaggaagtggaagcactactg-3’ | HA-R1  5’-gtagtctggcacgtcgtaaggata-3’ | 824 |
| *Rffl* | SD-F1  5’-gaaattccagacaagtttgttgttg-3’ | Rffl-E3-R1  5’acacgagtcctgtccgcctca-3’ | 565 |

**Supplemental Table 2.** TaqMan probes used for the quantification of gene expression in stress-resistant ES cell clones by RT-qPCR.

| Gene | Life Technologies  assay ID | Reference sequence | Exon  boundary | NCBI Location |
| --- | --- | --- | --- | --- |
| *Pigl* | Mm01334880_m1 | NM_001039536.2 | 1-2 | Chr 11: 62458460 - 62513900 |
| *Tiam1* | Mm00437065_m1 | NM_001145886.1 | 8-9 | Chr 16: 89787111 - 89974699 |
| *Rffl* | Mm03990772_m1 | NM_001007465.3 | 5-6 | Chr 11: 82803819 - 82871210 |

**Supplemental Table 3.** Generation of homozygous clone in culture by Blm knock-out

| **Heterozygous ES cell clone** | **No. of high G418 (2 mg/ml) resistant clone analyzed** | **No. of homozygous clone recovered** |
| --- | --- | --- |
| *Rffl ^PB^* / *Rffl ^+^* | 96 | 1 |
| *Pigl ^PB^*/ *Pigl ^+^* | 384 | 0 |
| *Tiam1 ^PB^* / *Tiam1 ^+^* | 384 | 0 |

Heterozygous ES cells were propagated under a dox-induced *Blm* deficient condition for about 15 doublings after which G418 selection was applied. Clones that arose from a single cell under G418 selection were picked and the somatic recombination events were analyzed by PCR.

**Supplemental Table 4.** Generation of mice

| **ES cell clone injected** | **No. of chimera recovered** | **Germline transmission *** |
| --- | --- | --- |
| *Pigl ^PB^*/ *Pigl ^+^* | 4 | Yes |
| *Tiam1 ^PB^* / *Tiam1 ^+^* | 2 | Yes |

* Germline transmission was confirmed by inheritance of the gene-trap allele detected by PCR genotyping.
