# Supplementary material for: Screening for stress-resistance mutations in the mouse
Source: Front Genet. 2014 Sep 8;5:310. doi: 10.3389/fgene.2014.00310 (PMC4157564; doi:10.3389/fgene.2014.00310)
Supplement: Supplementary file 2 [file Image1.PDF]

**Supplemental Figure 1.** The *Blm*$^{tet/tet}$ ES cells. (A) Gene targeting of the *Blm* allele. A targeting vector was constructed by taking a 2,421 bp DNA fragment immediately upstream of the *Blm* exon 2, amplified by PCR from mouse genomic DNA and cloned into the *Spe*I site of triTAUBi, a gene-switch plasmid kindly provided by Dr. John Adelman (Bond et al., 2000) as the left homologous arm. Similarly, a 2,780 bp DNA fragment encompassing the *Blm* exon 2 and downstream sequence was amplified and cloned into the *Not*I site of triTAUBi, forming the right homologous arm. Due to the hybrid nature of the EC7.1 ES cell line (F1 hybrid of C57BL/6 x 129X1/SvJ) (Chick et al., 2005), we generated two targeting vectors: one using C57BL/6 and another using 129 genomic DNA, to target both of the *Blm* alleles serially in EC7.1 ES cells. Homozygous targeted ES cells were identified by PCR using a primer annealing beyond the homologous arm and a primer annealing to the neo (the arrows indicate the primers used). The PCR products were digested with restriction enzymes to confirm identities (see insert). This *Blm*$^{tet/tet}$ ES cell line was designated "C9". (B) The mechanism of gene switch. In the *Blm*$^{tet}$ allele, the endogenous *Blm* promoter drives the expression of tTA protein, which in turns activates the downstream tetO5 CMV promoter to express Blm. Doxcycline, when presence, will bind to tTA proteins preventing them from activating the CMV promoter, so that *Blm* expression is turned off. (C) Transient switching-off of *Blm*. *Blm*$^{tet/tet}$ ES cells were treated with dox (1 µg/ml) at 0 h and later removed at 24 h. The transcript level of *Blm* was monitored by RT-qPCR prior to dox treatment (referenced as 100% expression) to 14 days after withdrawal of dox. (D) Western blot analysis. Blm protein was measured prior to dox treatment (0 h) and 8, and 24 h after dox treatment. The band with larger molecular weight was thought to be a nonspecific band associated with the nuclear fraction.

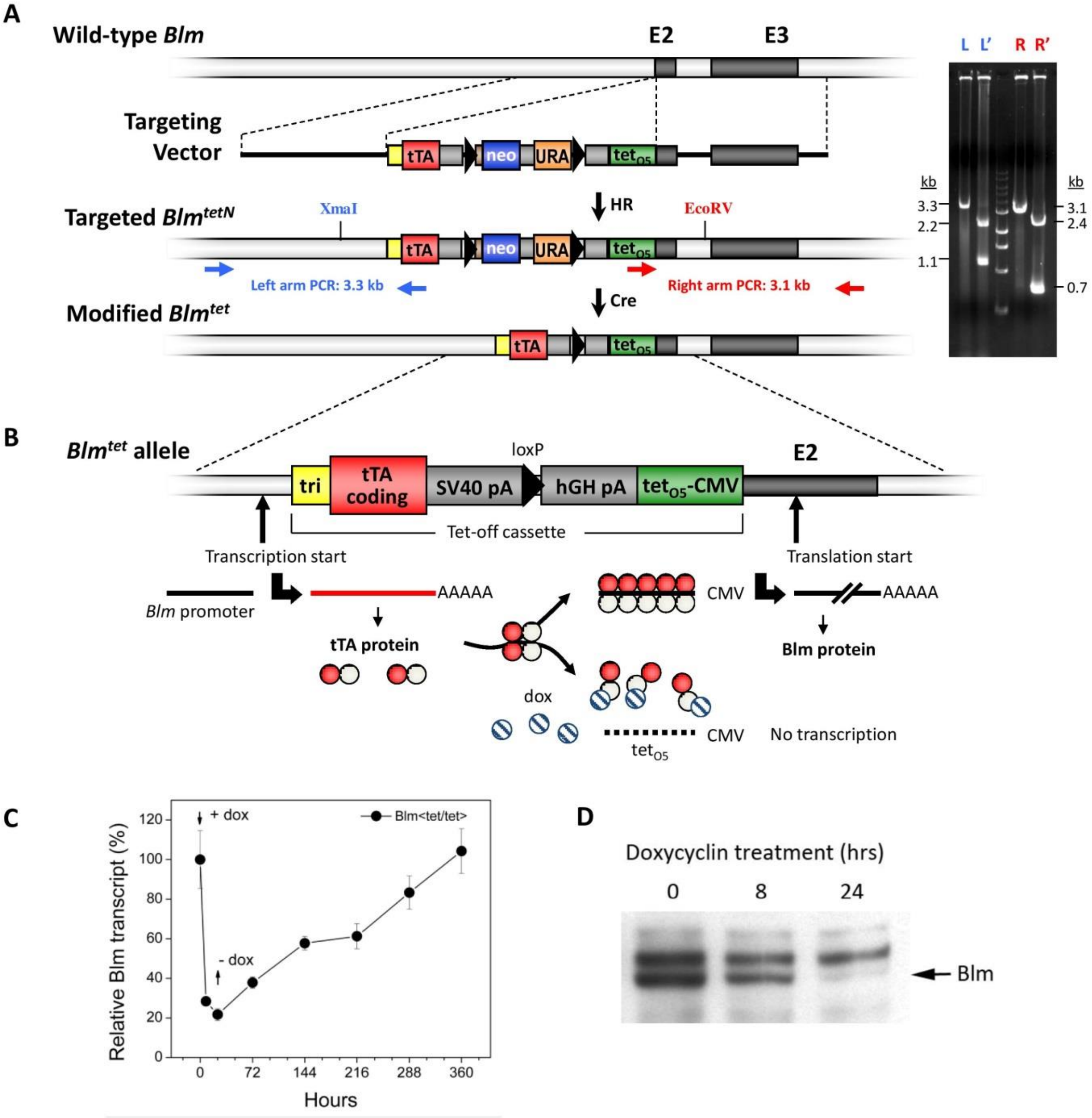
A
Wild-type Blm
E2
E3
Targeting Vector
tTA
neo
URA
tetO5
HR
Targeted BlmtetN
XmaI
EcoRV
Left arm PCR: 3.3 kb
Right arm PCR: 3.1 kb
Cre
Modified Blmtet
L
L'
R
R'
kb
3.3
2.2
1.1
3.1
2.4
0.7
B
Blmtet allele
tri
tTA coding
SV40 pA
loxP
hGH pA
tetO5-CMV
E2
Tet-off cassette
Transcription start
Translation start
Blm promoter
AAAAA
tTA protein
CMV
Blm protein
dox
No transcription
C
+ dox
- dox
Blm<tet/tet>
Relative Blm transcript (%)
Hours
D
Doxycyclin treatment (hrs)
0
8
24
Blm
